# Three-Dimensional Structure of Human NLRP10/PYNOD Pyrin Domain Reveals a Homotypic Interaction Site Distinct from Its Mouse Homologue

**DOI:** 10.1371/journal.pone.0067843

**Published:** 2013-07-04

**Authors:** Ming-Yuan Su, Chiao-I Kuo, Chi-Fon Chang, Chung-I Chang

**Affiliations:** 1 Institute of Biological Chemistry, Academia Sinica, Taipei, Taiwan, Republic of China; 2 Institute of Biochemical Sciences, College of Life Science, National Taiwan University, Taipei, Taiwan, Republic of China; 3 Genomics Research Center, Academia Sinica, Taipei, Taiwan, Republic of China; University of Iowa Carver College of Medicine, United States of America

## Abstract

NLRPs (Nucleotide-binding domain, leucine-rich repeat and pyrin domain containing proteins) are a family of pattern-recognition receptors (PRRs) that sense intracellular microbial components and endogenous stress signals. NLRP10 (also known as PYNOD) is a unique NLRP member characterized by a lack of the putative ligand-binding leucine-rich repeat domain. Recently, human NLRP10 has been shown to inhibit the self-association of ASC into aggregates and ASC-mediated procaspase-1 processing. However, such activities are not found in mouse NLRP10. Here we report the solution structure and dynamics of human NLRP10 pyrin domain (PYD), whose helix H3 and loop H2–H3 adopt a conformation distinct from those of mouse NLRP10. Docking studies show that human and mouse NLRP10 PYDs may interact differently with ASC PYD. These results provide a possible structural explanation for the contrasting effect of NLRP10 on ASC aggregation in human cells versus mouse models. Finally, we also provide evidence that in human NLRP10 the PYD domain may not interact with the NOD domain to regulate its intrinsic nucleotide hydrolysis activity.

## Introduction

The innate immune system in multicellular organisms has evolved to initiate the first line of host defense responses. Central to the innate immune system are several distinct families of extracellular, membrane-spanning, and intracellular receptor proteins known as the pattern-recognition receptors (PRRs), which play critical roles in detecting microbial intruders and environmental danger/stress signals, and in activating a wide spectrum of immune defense reactions. Several well known PRRs include the Toll-like receptors (TLRs), the peptidoglycan-recognition proteins (PGRPs), and the nucleotide-binding and leucine-rich repeat-containing receptors (NLRs) [1], [2], [3].

NLRs are a family of intracellular PRRs involved in sensing and regulating innate immune responses and apoptosis [4], [5], [6]. NLRs are multidomain proteins with a characteristic tripartite domain organization consisting of an N-terminal effector domain, which belongs to the death domain (DD) superfamily, a central nucleotide-binding oligomerization domain (NOD), and a C-terminal leucine-rich repeat (LRR) domain. The LRR domain responsible for recognition of diverse microbial ligands or environmental cues is present in almost all NLRs except for NLRP10. The N-terminal effector domain allows the NLRs to interact with downstream signaling proteins, which also share a common DD fold, via homotypic protein-protein interaction. These NLR effector domains consist of either a caspase activation and recruitment domain (CARD), a baculovirus inhibitor repeat domain (BIR), or in most cases a pyrin domain (PYD). Interaction of these effector domains with downstream signaling molecules is essential for NLRs to trigger and regulate diverse immune defense and inflammatory responses [4], [6].

NLRs with N-terminal PYDs, which consist of NLRP1–NLRP14 in human, represent the largest NLR subfamily. Several NLRPs, including NLRP1 and NLRP3, are known to participate in innate immune signaling by engaging the dual-domain adaptor protein ASC via homotypic PYD-PYD interactions. ASC in turn recruits procaspase-1 and activates its processing via homotypic CARD-CARD interaction. The multiprotein complex of NLRP-ASC-caspase-1, referred to as inflammasomes, orchestrates the activation of the proinflammatory caspases, which result in proteolytic processing of inflammatory cytokines [1]. Therefore, it is of great importance to understanding the structural basis of specific PYD-PYD interactions key to the assembly of inflammasomes. So far, structures are available for several isolated PYDs of several human NLRPs, as well as of human ASC and its inhibitor POP1/ASC2 [7], [8], [9], [10], [11], [12], [13]. However, the structure of a PYD-PYD complex has remained elusive.

NLRP10 (also known as PYNOD) is a unique member of the NLRP subfamily devoid of the ligand-binding leucine-rich repeat domain. Previous results have shown that human NLRP10 (hereafter huNLRP10) interacts with ASC and suppresses its aggregation, thereby inhibiting the processing of procaspase-1 and caspase-1–mediated IL-1β processing [14], [15]. Intriguingly, unlike human NLRP10, mouse NLRP10 (hereafter muNLRP10) does not inhibit ASC aggregation, nor is it able to inhibit procaspase-1 processing [14]. In line with these results, a recent mouse knock study shows that muNLRP10 does not act through an inflammasome to regulate caspase-1 activity nor that it regulates other inflammasomes; instead, NLRP10-deficient mice are defective in initiating adaptive immune responses [16]. These results collectively suggest that NLRP10 may play different roles in humans and in mice, which may in part due to its contrasting activity towards ASC in the two species. To understand the biochemical and structural basis of the function of NLRP10, we have determined the structure and dynamics of huNLRP10 PYD by NMR spectroscopy. Our results reveal significant structural difference between huNLRP10 and muNLRP10 PYDs. Docking studies suggest that huNRLP10 PYD and muNLRP10 PYD may use distinct binding sites to engage ASC PYD interaction, which may explain their contrasting effects on inhibiting ASC self-association in human versus mouse cells. In addition, using an MBP-tagged full-length huNLRP10 and a truncated construct without its PYD, we have analyzed the effect of the PYD on the nucleotide hydrolysis of NLRP10.

## Materials and Methods

### Protein Expression and Purification

Protein cloning, expression and purification of the PYD of huNLRP10/PYNOD (residues 1–100) were performed as recently described [17]. MBP-fusion proteins were constructed by cloning the gene fragments encoding the full-length huNLRP10 (Met^1^-Ile^655^) and huNLRP10ΔPYD (Arg^103^-Ile^655^) into pETM41 (EMBO collection), resulting in an N-terminal fusion with hexahistidine-maltose-binding protein. The MBP fusion proteins were overexpressed in *Escherichia coli* BL-21 (DE3) cells in LB medium containing additional 2 g/liter glucose by induction with 0.3 mM isopropyl-D-thiogalactoside (IPTG) at OD_600_ of 0.8 for 4 h at 30°C. Cell pellets were resuspended in lysis buffer (50 mM Tris-HCl pH 8.0, 500 mM NaCl, 20 mM imidazole and Roche EDTA-free protease inhibitor cocktail) and then lyzed by cell disruptor (EMULSIFLEX-C3 high pressure homogenizer, Avestin). After centrifugation at 35000×g, 4°C for 45 min, the supernatant was applied to a nickel-nitrilotriacetic acid column and washed with 20 mM imidazole. The protein fraction, eluted with 250 mM imidazole, was loaded onto a Superose 6 10/300 GL column equilibrated in 20 mM HEPES pH 7.5, 150 mM NaCl and 2 mM DTT.

### ATP/GTPase Assays

ATPase assays were performed using malachite green system to obtain kinetic parameters [18]. The reaction mixture containing 50 mM Tris-HCl (pH 8.0), 10 mM MgCl2, 0.1–3.2 mM ATP or GTP, and 3 µM NLRP10. The reaction was carried out by adding ATP or GTP to 20 µl of reaction mixture, incubated at 37°C for 20 min. Malachite green dye buffer of 160 µl (containing 0.0045% malachite green and 4.2% ammonium molybdate in 4N HCl) and 20 µl of 3.4% sodium citrate were then added. The mixtures were incubated at room temperature for 10 min for color development before measurement at OD_660_. Reactions containing no protein were performed to generate background readings of inorganic phosphate and were subtracted from the experimental results. The inorganic phosphate released was calculated based on the absorbance standard curve established by KH_2_PO_4_ standards. The kinetic parameters *K*_M_ and *k*_cat_ were calculated from the Lineweaver-Burk plot. All data were repeated at least three times.

### NMR Experiments

HuNLRP10 protein samples used for NMR studies were prepared in phosphate buffer saline (PBS) pH7.4 with 1 mM DTT and 10% (v/v) D_2_O as described [17]. All NMR experiments were carried out at 298K on Bruker Avance 600 or 800 MHz NMR spectrometers equipped with 5 mm triple resonance cryoprobe and Z-gradient. Protein backbone resonance assignments were based on standard triple resonance experiments: HNCA, HNCACB, CBCA(CO)NH, HNCO and HN(CA)CO. Aliphatic side-chain resonances were assigned based on HCCH-TOCSY, HCC(CO)NH and HBHA(CO)NH experiments. The data were acquired and processed using the software Topspin2.1 (Bruker, Germany) and further analyzed using CARA (developed by Kurt Wuthrich’s Group). ^1^H chemical shifts were externally referenced to 0 ppm of 2,2-dimethyl-2-silapentane-5-sulfonate, whereas ^13^C and ^15^N chemical shifts were indirectly referenced according to IUPAC recommendations [19]. NOE distance restraints were based on 3D ^15^N- and ^13^C-edited NOESY-HSQC experiments, both acquired with 100 ms mixing time. The steady-state heteronuclear ^15^N{^1^H}-NOE experiment was carried out at 600 MHz spectrometer in an interleaved manner, with and without proton saturation. Backbone ^1^H-^15^N residual dipolar couplings (RDCs) were measured at 800 MHz using^1^H-^15^N HSQC-IPAP pulse sequence [20].

### NMR Structure Calculation and Relaxation Analysis

Semi-automated NOE cross-peak assignments for distance restraints were performed using CANDID module in software CYANA3.0 [21], [22] and manually checked for correctness in iterative manner. Dihedral angle restraints were predicted from the backbone chemical shifts using program TALOS [23]. In addition, RDC data collected in the Pf1-aligned phage (ASLA biotech, ca. 10 mg/ml) were provided as input to CYANA for structure refinement calculation. Errors in backbone heteronuclear ^15^N{^1^H}-NOE were expressed as the standard deviation of three pairs of repeated ^15^N{^1^H}-NOE experiments.

### Protein-protein Docking

Docking analysis between NLRP10 PYD and ASC PYD was performed using the Cluspro 2.0 server (http://cluspro.bu.edu) [24]. For docking studies of mouse ASC PYD, a homology model was built based on human ASC PYD (PDB code 1UCP) using the SWISS-MODEL server (http://swissmodel.expasy.org) [25]. No residue constraints were specified as inputs for docking calculations. The huNLRP10 and muNLRP10 PYDs were assigned as the receptors in the docking studies.

## Results and Discussion

### NLRP10 has Both ATPase and GTPase Activities

NLRP10 is the only NLRP member containing an N-terminal PYD and a C-terminal NOD. We have sought to express and purify this unique NLRP for structural and functional analyses as a first step towards dissecting its action mechanism. Recombinant huNLRP10 or a truncated construct containing only the NOD formed inclusion bodies when expressed in *E. coli*. However, huNLRP10 could be expressed as a soluble protein in fusion with the maltose-binding protein (MBP) at the N-terminus. As the C-terminal NOD exhibits conserved sequence features of the AAA+ (ATPases Associated with diverse cellular Activities) family domains, we generated full-length MBP-huNLRP10 as well as a truncated MBP-huNLRP10ΔPYD, containing only the NOD, to examine their nucleotide hydrolysis activities. We found that both constructs exhibited moderate ATPase and GTPase activities **(** **Table 1** **)**. While the *K*_M_ values of the two constructs were comparable, the *k*_cat_ of the MBP-huNLRP10ΔPYD was slightly higher than MBP-huNLRP10. The enzymatic activity of both constructs appeared to be more efficient towards ATP than GTP, as judging from the higher *k*_cat_/*K*_M_ value for ATP. Although the *k*_cat_ for ATP hydrolysis of huNLRP10ΔPYD was almost two-fold higher than huNLRP10, which may indicate weak intramolecular interaction between the PYD and NOD modules, we did not observe any interaction between these two domains by size-exclusion chromatography or pull-down assay using purified NLRP10 PYD and MBP-huNLRP10ΔPYD (data not shown). Overall, these results suggest that the PYD domain may not be involved in regulation of the nucleotide hydrolysis activity of NLRP10.

**Table 1:** Nucleotide hydrolysis kinetics of MBP-tagged huNLRP10 constructs.

Parameters	*K*_M_ (µM)	*k*_cat_ (m^−1^)	*k*_cat_ */K*_M_	*K*_M_ (µM)	*k*_cat_ (m^−1^)	*k*_cat_ */K*_M_
Nucleotides	ATP			GTP		
huNLRP10	169.3±7.3	0.3497±0.0078	0.0021	295.6±11.0	0.3374±0.0061	0.0011
huNLRP10ΔPYD	201.9±1.9	0.6634±0.0036	0.0033	193.7±17.1	0.3755±0.0193	0.0019

### Overall Structure of huNLRP10 PYD

**Figure 1:**
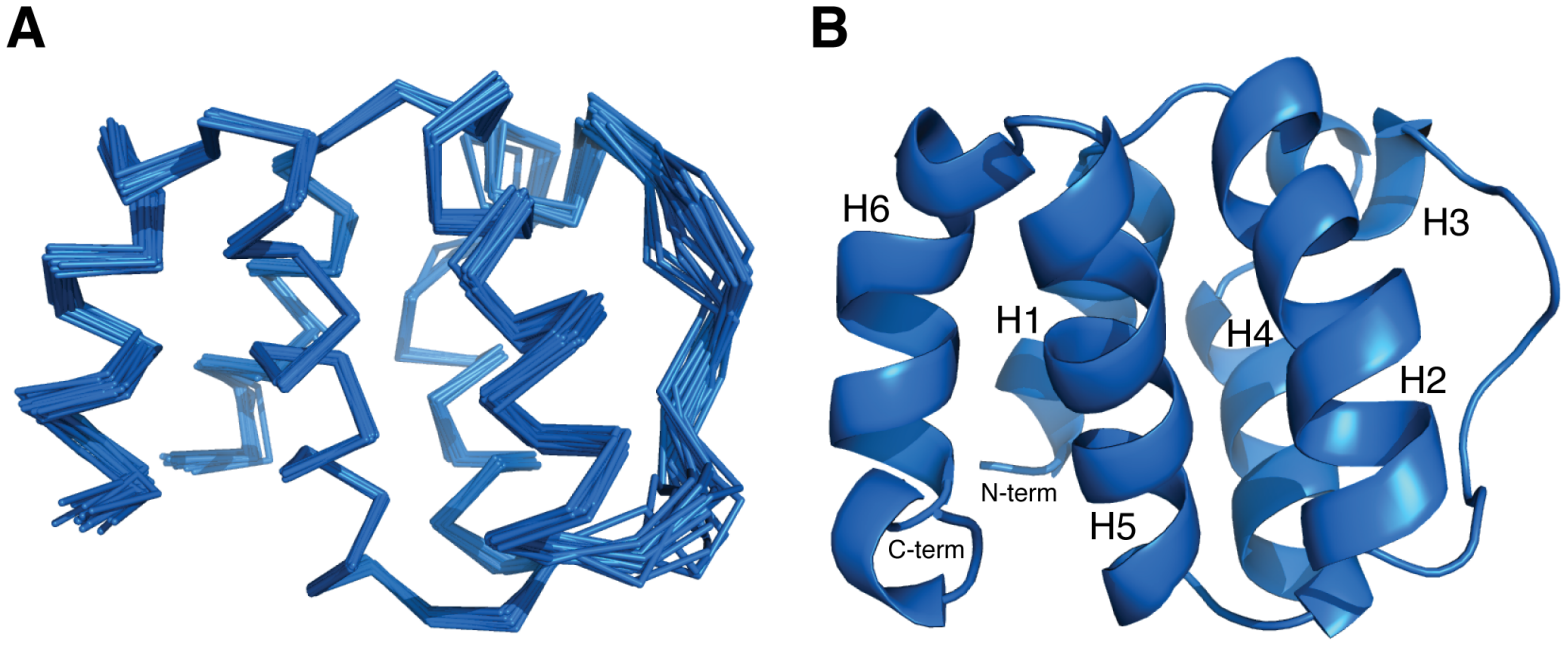
NMR structure of huNLRP10 PYD. (A) Ensemble of the 20 lowest-energy CYANA conformers, superimposed on the backbone atoms of the six helices, representing the structure of huNLRP10 PYD. (B) Ribbon representation of the lowest-energy structure in the same orientation ass in (A). The six helices, as well as the N-and C-termini, are labeled.

**Figure 2:**
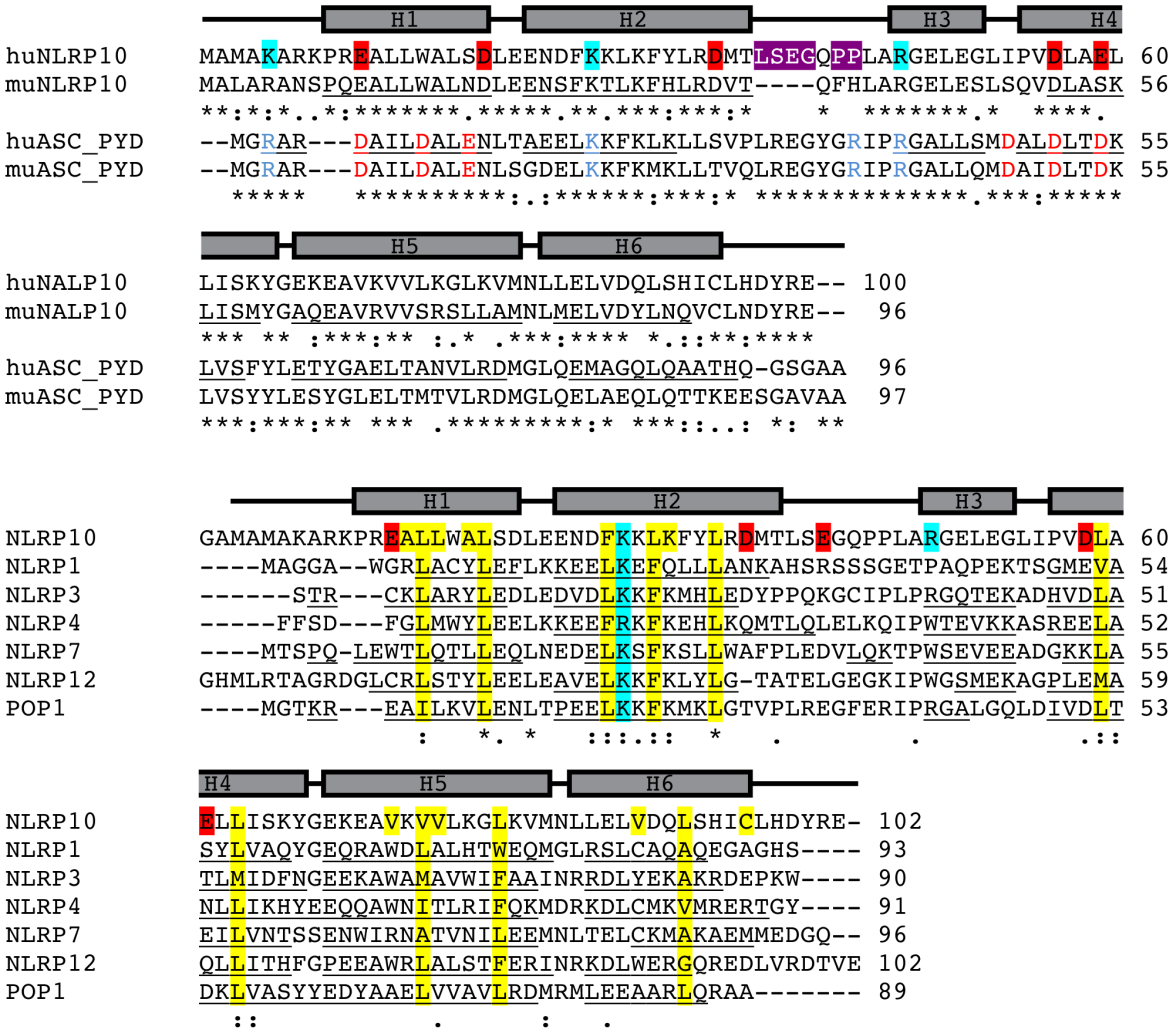
Sequence alignment of huNLRP10 PYD with other PYDs. (A) Sequence alignment of huNLRP10 and muNLRP10s and ASCs. Experimentally determined -helices of huNLRP10 PYD are depicted by gray rectangles above the sequences. Helical residues of all other PYDs with known structures are underlined. The tetrapeptide insertion and replaced dipeptide sequences in the H2–H3 loop of huNLRP10 are shaded in violet. Acidic and basic residues determined previously in ASC PYD to be critical for self-association and interacting with other PYDs are colored in red and blue, respectively [30], [31]; the corresponding acidic and basic residues, located on the H1–H4 and H2–H3 surfaces of huNLRP10 PYD are shaded in red and cyan, respectively. Residues forming the hydrophobic core are shaded in yellow. Symbols below the aligned sequences are: “*” for identical residues; “:” for conserved residues; “.” for semi-conserved residues.

HuNLRP10 PYD was highly soluble in physiological pH when overexpessed as a standalone protein in *E. coli*. HuNLRP10 PYD in pH 7∼8 was a monomer as judging by size-exclusion chromatography. Hence, we performed all NMR measurements of huNLRP10 PYD in phosphate buffered saline in pH 7.4 [17]. The tertiary structure of huNLRP10 PYD was determined from 947 distance restraints assigned by CYANA and 157 torsion angles (78ψ, 79φ), and the orientation of secondary structure were further refined using 45 RDCs (correlation coefficient 0.99). The superposition of the resultant family of 20 best structures of the huNLRP10 PYD is shown in Figure 1A. The structural statistics is given in **Table 2**. The average root mean square deviations (RMSD) for backbone atoms are 0.27±0.08 Å for the well-defined secondary structure regions and 0.49±0.12 Å for the segment over residues 10–96. The quality of these structures is good as judged by the fact that most of the backbone torsion angles for non-glycine and non-proline residues fall in either the most favorable (80.5%) or the allowed regions (19.5%) of the Ramachandran plot, when calculated with the PROCHECK_NMR program [26]. The huNLRP10 PYD structure contains six α-helices (H1–H6) connecting by loops, which adopts a canonical DD fold characterized by an anti-parallel six-helical bundle **(** **Figure 1** **)**. The helical bundle of huNLRP10 PYD is stabilized by Ala14, Leu15, Leu16, Ala18, and Leu19 of H1; Phe27, Leu30, Lys31, and Leu34 of H2; Leu59 and Leu63 of H4; Val73, Val75, Val76, and Leu80 of H5; Val89, Leu92, and Cys96 of H6, which constitute a central hydrophobic core conserved in all DD members **(** **Figure 2** **)**. The helix H3, which is not present in NLRP1/NALP1, is anchored to the hydrophobic core mainly through Leu51, which interacts with Phe27 from H2. The extended H2–H3 loop, comprising Leu39-Ala47, is loosely packed against the H2–H4 interface; its conformation is maintained by Leu39 and Leu46 through hydrophobic interactions with H2 and the conserved hydrophobic core, respectively.

**Table 2:** Summary of Structural Constraints and Structural Statistics.

All NOE distance restraints^a^	947
Sequential (i, i+1)	530
Medium-range	267
Long-range (|i-j|>4)	150
Total dihedral angles	
Φ(deg) b	79
Ψ(deg)^b^	78
Average Target Function (Å^2^)	12.07
RMSD deviation for residues 10–96	
Average backbone to mean	0.49±0.12
Average heavy atom to mean	1.06±0.13
RMSD deviation for secondary structure^c^	
Average backbone to mean	0.27±0.08
Average heavy atom to mean	0.89±0.07
Structure analysis as defined by PROCHECK	
Residues in most favored regions (%)	80.5
Residues in additional allowed regions (%)	19.4
Residues in generously allowed regions (%)	0.1
Residues in disallowed regions (%)	0.0

a Final NOE restraints defined by program CYANA.

b Dihedral angles predicted from program TALOS.

c Residues 12–21,27–38, 48–53, 56–66, 69–83 and 85–96.

### Backbone Dynamics of NLRP10 PYD

**Figure 3:**
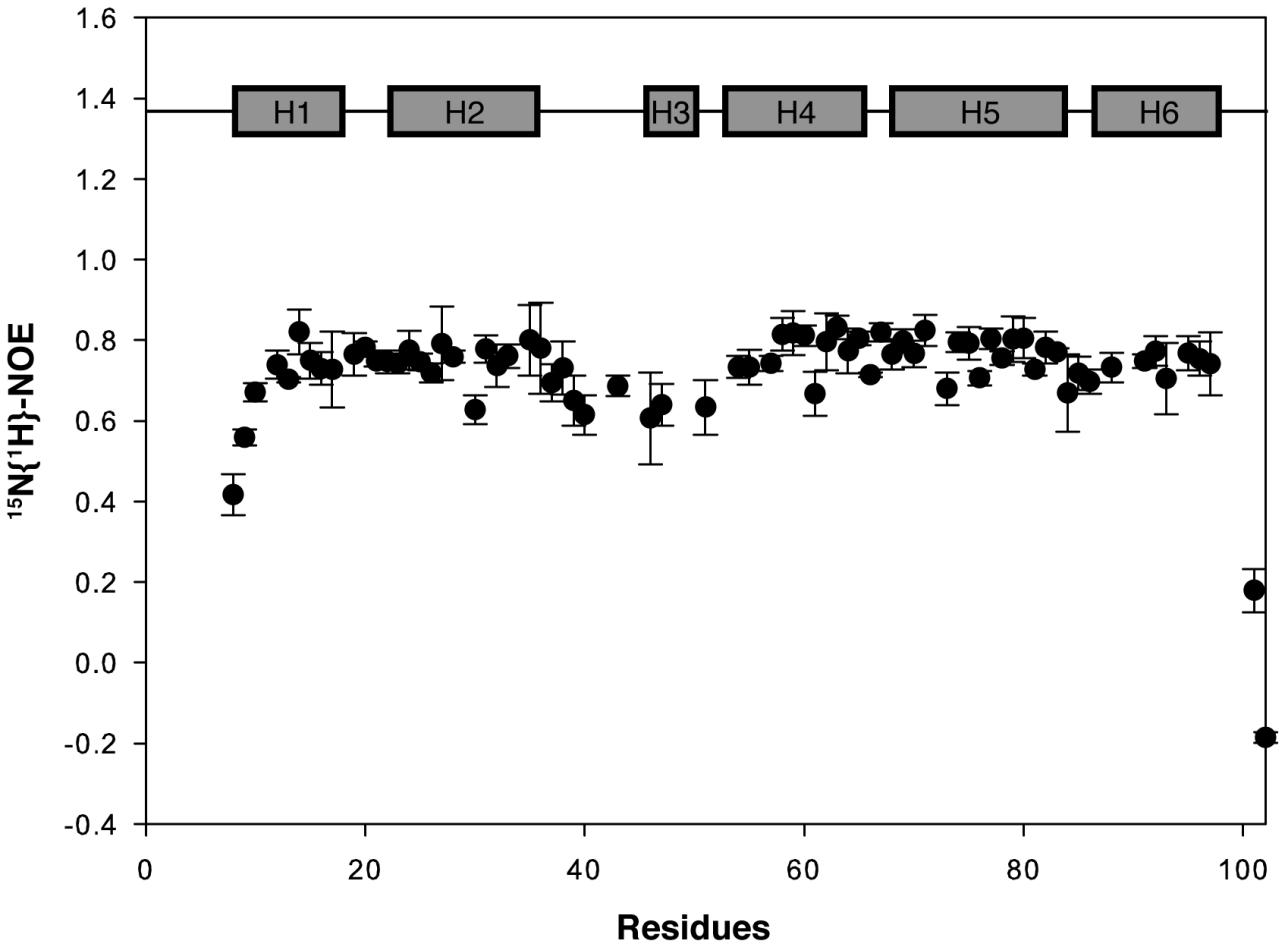
Backbone dynamics of huNLRP10. NMR relaxation data of backbone amide (^15^N) measured as heteronuclear Overhauser values (^15^N{^1^H}- NOE) showing local motions on a fast time scale (ps to ns) of the residues in the H2–H3 loop and helix H3 regions.

**Figure 4:**
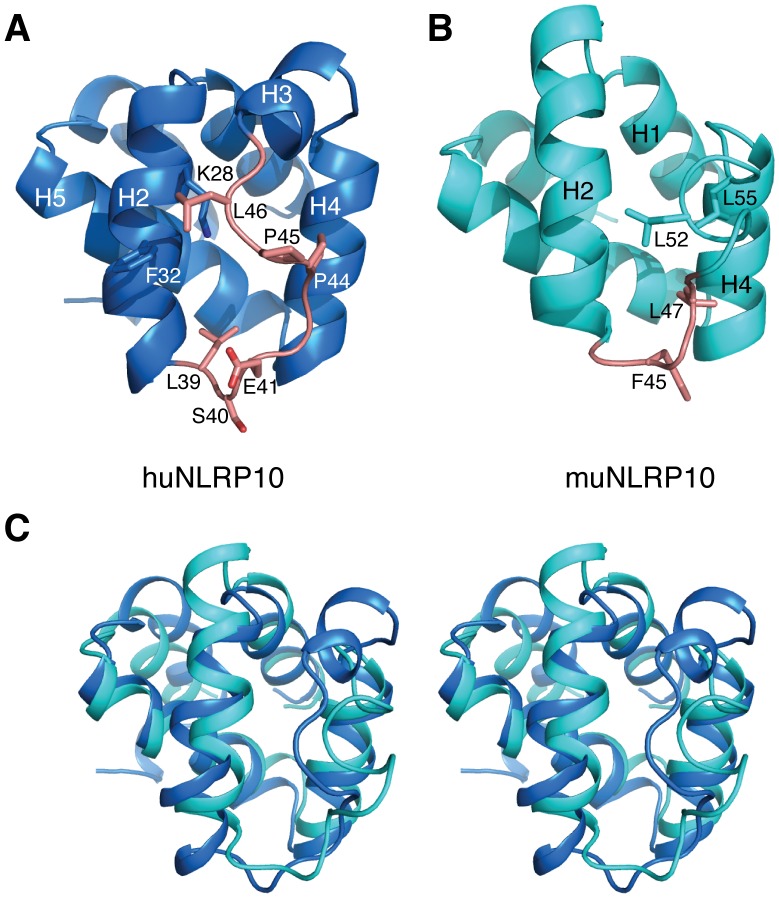
Structures of huNLRP10 and muNLRP10 PYDs. (A) Ribbon representation of huNLRP10 PYD with the H2–H3 loop highlighted in orange. (B) Ribbon representation of muNLRP10 PYD (PDB code 2DO9), in identical orientation as huNLRP10 PYD shown in (A), with the highlighted region corresponding to the H2–H3 loop of the human homologue. Residues in sticks are labeled and discussed in main text. (C) Stereo view showing the superimposed structures of huNLRP10 PYD (blue) and muNLRP10 PYD (cyan).

We investigated the backbone dynamics in huNLRP10 PYD by heteronuclear ^15^N{^1^H}-NOE measurements. These auto-correlated ^15^N-relaxation data, which report backbone motions on fast (picosecond–nanosecond) timescale, show a small increase in fast timescale dynamics in both the H2–H3 loop and the H3 helix regions **(** **Figure 3** **)**. Thus, the detected backbone dynamics in the H2–H3 loop of huNLRP10 appear to be similar to those reported for NLRP12 [8]. By comparison, the H2–H3 loop in NLRP7, ASC, and POP1 showed the same degree of rigidities as the neighboring helices [10], [11], [27]; on the other hand, the corresponding regions of NLRP1 showed substantial structural disorder [13]. Although the backbone dynamics of muNLRP10 have not been published, it is possible that the two homologues may exhibit different backbone flexibility on the H2–H3 loop based on their marked sequence and structural variances **(** **Figure 4** **)** (discussed below).

### Structural Comparison of huNLRP10 PYD with muNLRP10 PYD and ASC PYD

HuNLRP10 PYD shares significantly higher sequence identity (42%) with its mouse homologue than with all other NLRP PYDs (20–30%) whose three-dimensional structures are known to date **(** **Table 3** **)**. However, the H2–H3 loops of huNLRP10 and muNLRP10 PYDs are drastically different in not only their length but also the composing amino acids **(** **Figure 2** **)**. In fact, structural similarity analysis using the Dali server [28] has revealed that huNLRP10 PYD is even distinctively more different from muNLRP10 PYD (PDB code 2DO9 [29]) than from most other PYDs **(** **Table 3** **)**. The major structural difference is localized to the H2–H3 loop and the H3 helix. In huNLRP10 PYD, the H2–H3 loop is in an extended conformation maintained in part by Leu39, which forms part of the hydrophobic core, and by Leu46, which makes van der Waals contacts with Lys28 and Phe32 of H2 **(** **Figure 4** **)**. By contrast, the missing of four residues (Leu39-Ser40-Glu41-Gly42) found in its human homologue and the replacement of the dipeptide sequence Pro-Pro by Phe45-His46 in muNLRP10 PYD result in a twisted loop connecting helices H2 and H4, where helix H3, which is present in huNLRP10, is deformed. The twisted H2–H3 loop in muNLRP10 PYD is anchored to helices H2 and H4 via Phe45, Leu47, and Leu52, and Leu55 by hydrophobic interactions **(** **Figure 4** **)**.

**Table 3:** Structural similarity of huNLRP10 PYD to other PYDs.

PYDs	Z-score	Identity (%)	r.m.s.d. (Å)	PDB code
ASC	8.3	26	2.8	2KN6
NLRP3	8.1	27	2.4	3QF2
NLRP7	7.9	24	2.7	2KM6
POP1	7.7	30	2.6	2HM2
NLRP4	7.7	24	2.3	4EWI
NLRP12	7.2	28	3.3	2L6A
muNLRP10	6.8	42	4.2	2DO9
NLRP1	5.9	22	2.4	1PN5

Structural comparison of PYDs using Dali [28]; mNLRP10 means mouse NLRP10; r.m.s.d. means root mean square deviation.

Based on all available PYD structures it has been established that helix H3 and the preceding loop H2–H3 are highly variable in terms of their sequences and conformations. In addition, both crystallographic and NMR dynamics data have revealed that these two regions are in general more flexible than other parts of the structure; also they exhibit distinct backbone dynamics properties in different PYDs [7], [8], [9], [10]. In the extreme case, NLRP1 PYD completely lacks helix H3 and instead forms a long flexible linker between helices H2 and H4 [13]. As helices H2–H3 forms a charged surface demonstrated to be critical for homotypic or heterotypic PYD interactions [8], [30], [31], it has been postulated that the flexibility and conformation of the distinct H2–H3 surface may contribute to binding of each NLRP member to specific effector proteins. Here, comparison of loop H2–H3 and helix H3 regions from huNLRP10 and muNLRP10 PYDs further highlights the unique sequence and conformational variability in these two regions. The drastic structural difference between the two NLRP10 PYDs is mainly caused by a tetrapeptide insertion present in human but not in mouse homologues. Functional difference caused by structural alteration due to similar insertions between two homologous proteins with high sequence identity has been observed for *Drosophila* PGRPs [32], [33]. Interestingly, several recent studies have revealed contrasting activities for NLRP10 in human cells and in mouse models. These works showed that huNLRP10 binds to ASC and, by suppressing ASC aggregation, it inhibits the processing of procaspase-1 and caspase-1–mediated IL-1β processing; however, unlike huNLRP10, muNLRP10 does not inhibit ASC aggregation, nor is it able to inhibit procaspase-1 processing [14], [15]. It should be noted that human and mouse ASCs share a high sequence identity (76.5%), with no insertions occurred in all helices; all the residues identified in human ASC important for ASC PYD interactions are conserved in the mouse protein **(** **Figure 2** **)** [30], [31].

### Docking Studies of huNLRP10 PYD and muNLRP10 PYD with ASC PYD

**Figure 5:**
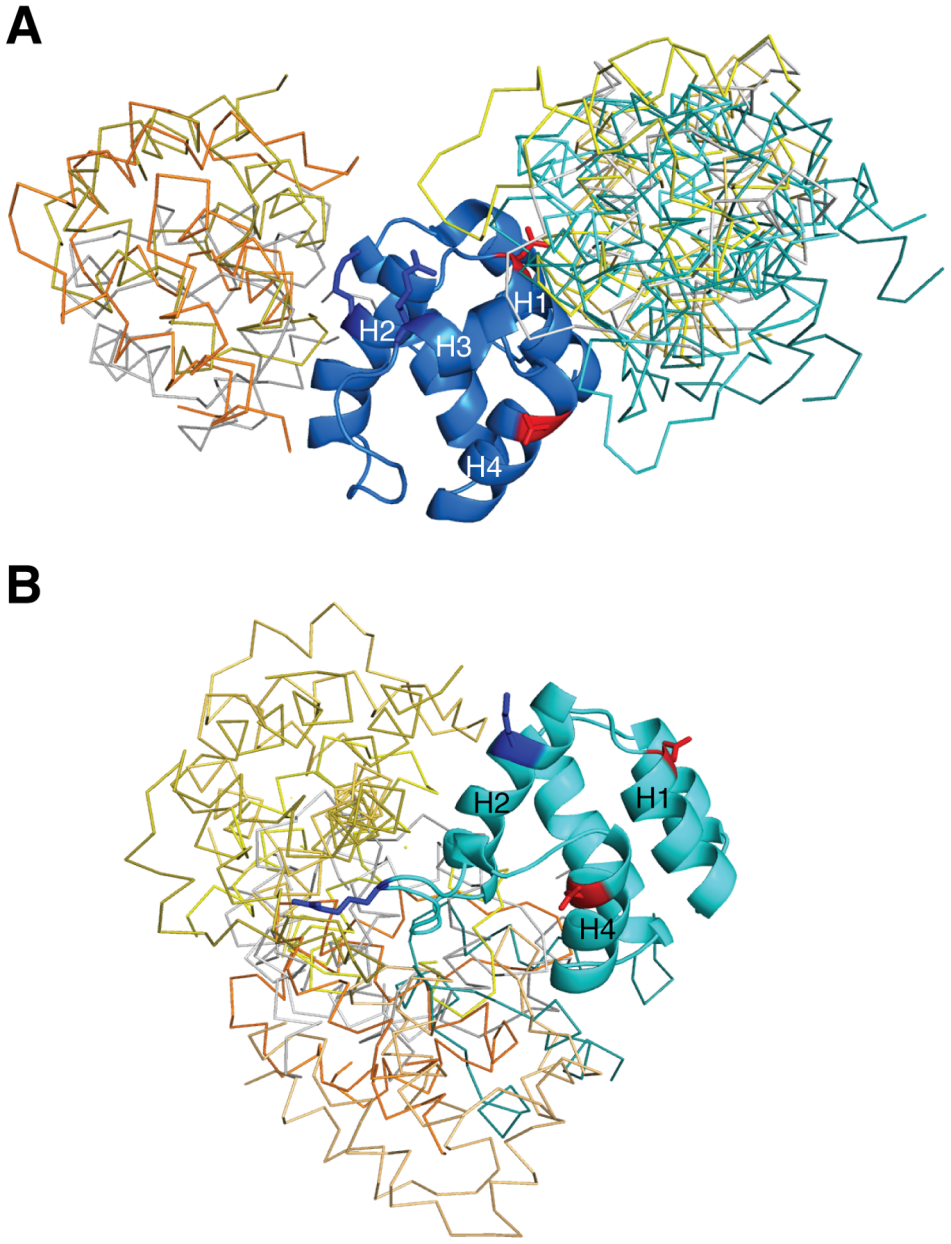
Docking analysis of huNLRP10 and muNLRP10 PYDs with ASC PYD. Superposition of the top 10 docking models of the huNLRP10 PYD-ASC PYD complex (A) and of the muNLRP10 PYD-ASC PYD complex (B) predicted by ClusPro (http://nrc.bu.edu/cluster). Note that none of the top 10 models of the mouse complex involves the H1–H4 surface of muNLRP10 PYD. The docked huASC and muASC PYD models are shown in backbone traces with different colors. The huNLRP10 and muNLRP10 PYDs, defined as the receptors for docking, are rendered in marine- and cyan-colored ribbons, respectively; the conserved basic and acidic residues shown in blue and red sticks, respectively, were identified previously to be important in ASC PYD for self-association.

**Figure 6:**
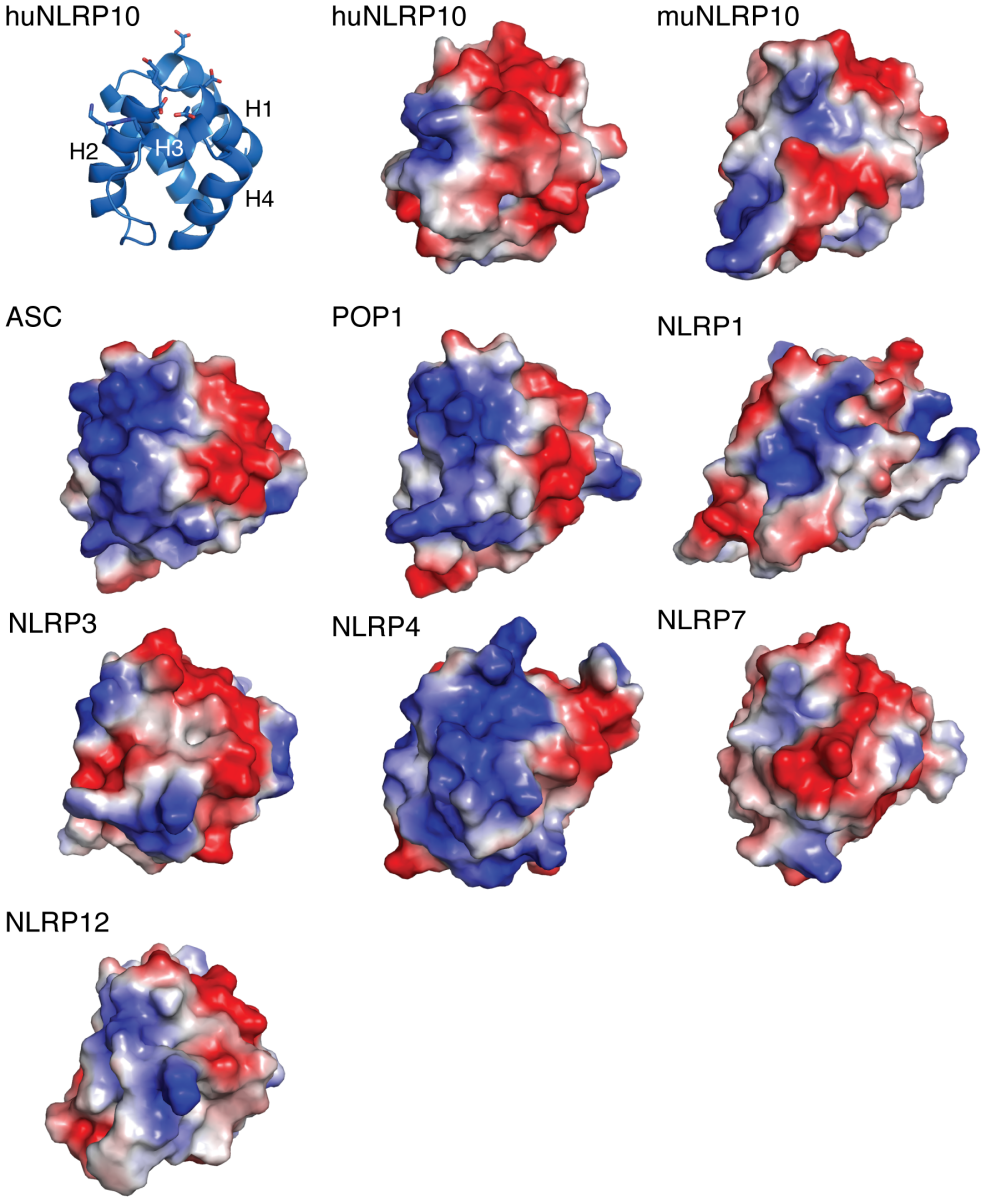
Surface electrostatic potential of the PYDs of huNLRP10, muNLRP10, and other PYDs. All surface representations of the PYDs are shown in the same orientation as the ribbon model of huNLRP10 on the upper left, with the H2–H3 and the H1–H4 surfaces on the left side and the right side, respectively, of each model. Positive surface charge is colored in blue; negative surface charge is colored red; and neutral surface is in white. The exposed basic and acidic residues on the two surfaces are shown as sticks in the ribbon representation of huNLRP10.

Both human and mouse NLRP10 have been shown to co-localized with ASC, suggesting that both may be capable of binding ASC [14]. To understand how the sequence and structural differences between huNLRP10 and muNLRP10 PYDs may translate into their contrasting activity on inhibiting ASC self-association, we performed docking studies of huNLRP10 and huASC PYDs using the ClusPro protein-protein docking server [24]. The results show that huNLRP10 PYD may engage interaction with ASC PYD through two distinct binding surfaces, one formed by helices H2 and H3 and the other by helix H1, loop H3–H4, and helix H4 **(** **Figure 5A** **)**. Importantly, these two binding surfaces have been mapped previously by systematic mutagenesis analysis as the two binding sites mediating self-association of ASC into aggregates [30]. By contrast, docking analysis between muNLRP10 PYD and a homology model of muASC PYD shows that muNLRP10 PYD uses primarily only the H2–H3 surface for ASC interaction **(** **Figure 5B** **)**. These docking results suggest that huNLRP10 PYD may use the similar binding sites to compete for interaction with ASC PYD, thereby preventing the self-association of ASC into aggregates. On the other hand, muNLRP10 PYD may not use its H1–H4 surface for potential ASC interaction, presumably due to a less-contiguous acidic surface charge distribution **(** **Figure 6** **)**. As a result, muNLRP10 PYD may only bind to oligomerized ASC without interfering its self-association. Overall, these results are in line with the contrasting activities of huNLRP10 and muNLRP10 on the inhibition of ASC signaling found in previous cellular and animal studies [14].

### Surface Charge Comparison of NLRP10 PYD with Other PYDs

Homotypic PYD interactions play a major role in assembly of inflammasome complexes, which induce an inflammatory response through activation of procaspase-1 and lead to the proteolytic activation of the pro-inflammatory cytokines IL-1β and IL-18 [1]. Little is known for the structural basis of these molecular interactions; however, recent biochemical analyses have revealed two distinct binding sites important for PYD-PYD interactions [30], [31]. These two sites were mapped to the negatively charged H1–H4 and the positively charged H2–H3 surface residues on ASC PYD, which were shown to be critical for self-association of ASC and for interaction of ASC with NLRP3 and POP1. As shown in **Figure 6**, the surface electrostatic potential of these two sites on huNLRP10 PYD is in a similar charge arrangement as those exhibited on the PYDs of ASC, POP1, and NLRP4. By contrast, the surface charge distribution and arrangement of huNLRP10 PYD is remarkably different from muNLRP10 PYD. The surface electrostatics of the latter is in fact more similar to NLRP7 PYD **(** **Figure 6** **)**. Recent crystal structures of multimeric DD complexes, including the MyDDosome, the PIDDosome and the Fas/FADD-DISC, have revealed an emerging theme for oligomerization of members of the DD superfamily, which requires multiple interaction sites on each DD component, as well as successive recruitment of the interacting components [34]. It is likely that a similar multimeric platform may also be assembled via homotypic PYD-PYD interactions, which for example are critical for the formation of the ASC aggregates, as well as the NLRP1 and NLRP3 inflammasome complexes. Other NLRPs, such as NLRP10, may execute their specific biological functions by modulating the formation of these well known multimeric platforms through specific interaction sites on their PYDs; these interactions may either inhibit or strengthen the oligomerization of the signaling complexes. A structure of a PYD-PYD complex will be indispensible to help define the molecular mechanism for this important type of homotypic domain-domain interaction.
